# Biflavone Ginkgetin, a Novel Wnt Inhibitor, Suppresses the Growth of Medulloblastoma

**DOI:** 10.1007/s13659-015-0056-4

**Published:** 2015-03-29

**Authors:** Zhen-Nan Ye, Mu-Yuan Yu, Ling-Mei Kong, Wei-Hua Wang, Yuan-Feng Yang, Jie-Qing Liu, Ming-Hua Qiu, Yan Li

**Affiliations:** 1 State Key Laboratory of Phytochemistry and Plant Resources in West China, Kunming Institute of Botany, Chinese Academy of Sciences, Kunming, 650201 Yunnan China; 2 University of Chinese Academy of Sciences, Beijing, 100049 China

**Keywords:** Wnt signaling, Medulloblastoma, Inhibitor, Biflavone, Ginkgetin

## Abstract

Medulloblastoma (MB) is a form of malignant brain tumor that predominantly arises in infants and children, of which approximately 25 % is due to upregulation of canonical Wnt pathway with mainly mutations in *CTNNB1*. Therefore, Wnt inhibitors could offer rational therapeutic strategies and chemoprevention for this malignant cancer. In our present study, we undertook a screening for antagonists of Wnt signaling from 600 natural compounds, and identified Ginkgetin, a biflavone isolated from *Cephalotaxus fortunei* var. *alpina*. Ginkgetin inhibited Wnt pathway with an IC_50_ value around 5.92 μM and structure–activity relationship analysis suggested the methoxy group in Ginkgetin as a functional group. Biflavone Ginkgetin showed obvious cytotoxicity in Daoy and D283 MB cells. Cell cycle analysis by flow cytometry showed that Ginkgetin induced efficiently G_2_/M phase arrest in Daoy cells. Further mechanism studies showed that Ginkgetin reduced the expression of Wnt target genes, including Axin2, cyclinD1 and survivin in MB cells. The phosphorylation level of *β*-catenin also decreased in a time- and concentration-dependent manner. Collectively, our data suggest that Ginkgetin is a novel inhibitor of Wnt signaling, and as such warrants further exploration as a promising anti-medulloblastoma candidate.

## Introduction

Medulloblastoma (MB) is a form of pediatric brain cancer for which no satisfactory treatments exist, making up 20 % of all primary central nervous system (CNS) tumor in patients under 19 [1]. MB was first distinguished from glioma by Bailey and Cushing in 1925 [2]. The tumor arises from granule-cell progenitors locating in the neuroectodermal of the cerebellum, a germinal zone undergoing active proliferation during embryogenesis [3, 4]. Human tumor samples and murine transgenic models suggest that cerebellar stem cells with certain genetic alterations can be transformed into MB [5]. The current World Health Organization (WHO) classification (2007) has identified MB as a kind of embryonal tumors [6]. Therefore, the cancer stem-like cell (CSC) subpopulations, also known as brain tumor-initiating cells (BTICs), demonstrate a potential therapeutic target in MB.

Wnt signaling and other signaling pathways, such as SHH signaling, which are vital regulators of the self-renewal and maintenance of stem cells, are closely involved in the tumorigenesis of MB. The activated Wnt pathway in the tumorigenesis of MB was first recognized in the Turcot syndrome, a familial cancer syndrome [7]. Mutations in several Wnt members have been identified since then, including *CTNNB1* [8] activating, *APC* [9] and *Axin* [10] inactivating, which account for 25 % of sporadic MB [11]. A recent study has shown that loss of chromosome 6 was also correlated with this subtype [12]. Genomic alterations of SHH signaling mainly harbor in *PTCH1* [13]*, SUFU* [14] and *SMO* [15]. Based on the gene mutations described above, MB was classified into wingless (WNT; Group 1), sonic hedgehog (SHH; Group 2), and the other two groups including Group 3 and Group 4 [16, 17], totally four distinct molecular variants. Although the histological and molecular characteristics of the four subtypes MB are different, the current treatment strategy is almost the same, including surgical resection, high-dose chemotherapy and irradiation of the entire neuraxis depending on the patient’s age [18]. The 5-year survival rates have improved in the past decades due to recognition of the specific subtypes and advances in risk-directed treatments [19]. Nevertheless, those who survive often suffer from neurologic, endocrinologic and social sequelae as a consequence of therapy. Thus, there remains a great demand for new targeted therapeutic approaches, which would be invaluable in relieving the multiple adverse effects of the traditional approaches and improve patients’ survival and the quality of life.

Since Wnt signaling is over-activated in MB, inhibition of Wnt signaling has been proved to be a potential approach for the prevention of the tumor. Transfection with DKK1, a Wnt antagonist, in D283 cells, suppressed colony formation and induced apoptosis [20]. NCTD, a demethylated cantharidin analog, impaired Wnt signaling, attenuated the attachment ability of MB cell lines and inhibited the Daoy xenograft animal model [21]. OSU03012 inhibited PI3K/Akt and Wnt cross-talk, decreased levels of *β*-catenin and Wnt target genes, and suppressed D283 xenografts growth [22]. Urokinase plasminogen activator receptor (uPAR) has a mutual regulatory mechanism with Wnt signaling. Swapna et al. reported that pU/pUM constructs which were designed to knockdown uPAR, suppressed Wnt signaling, and reduced spheroids formation and migration, in vitro and in vivo [23].

In the present study, we performed a screening for Wnt inhibitors and identified compound **1** (Ginkgetin), a biflavone, as a new antagonist. We evaluated the potential inhibitory activity of Ginkgetin on MB and found that Ginkgetin efficiently suppressed the growth of MB cancer cells. Ginkgetin induced the G_2_/M phase arrest and inhibited the Wnt signaling in Daoy cells. Our data suggested that Ginkgetin was a novel inhibitor of Wnt signaling, and might deserve further exploration as an anti-medulloblastoma agent.

## Results and Discussion

### Identification of Biflavone Ginkgetin as a Potent Inhibitor of Wnt Pathway

**Fig. 1:**
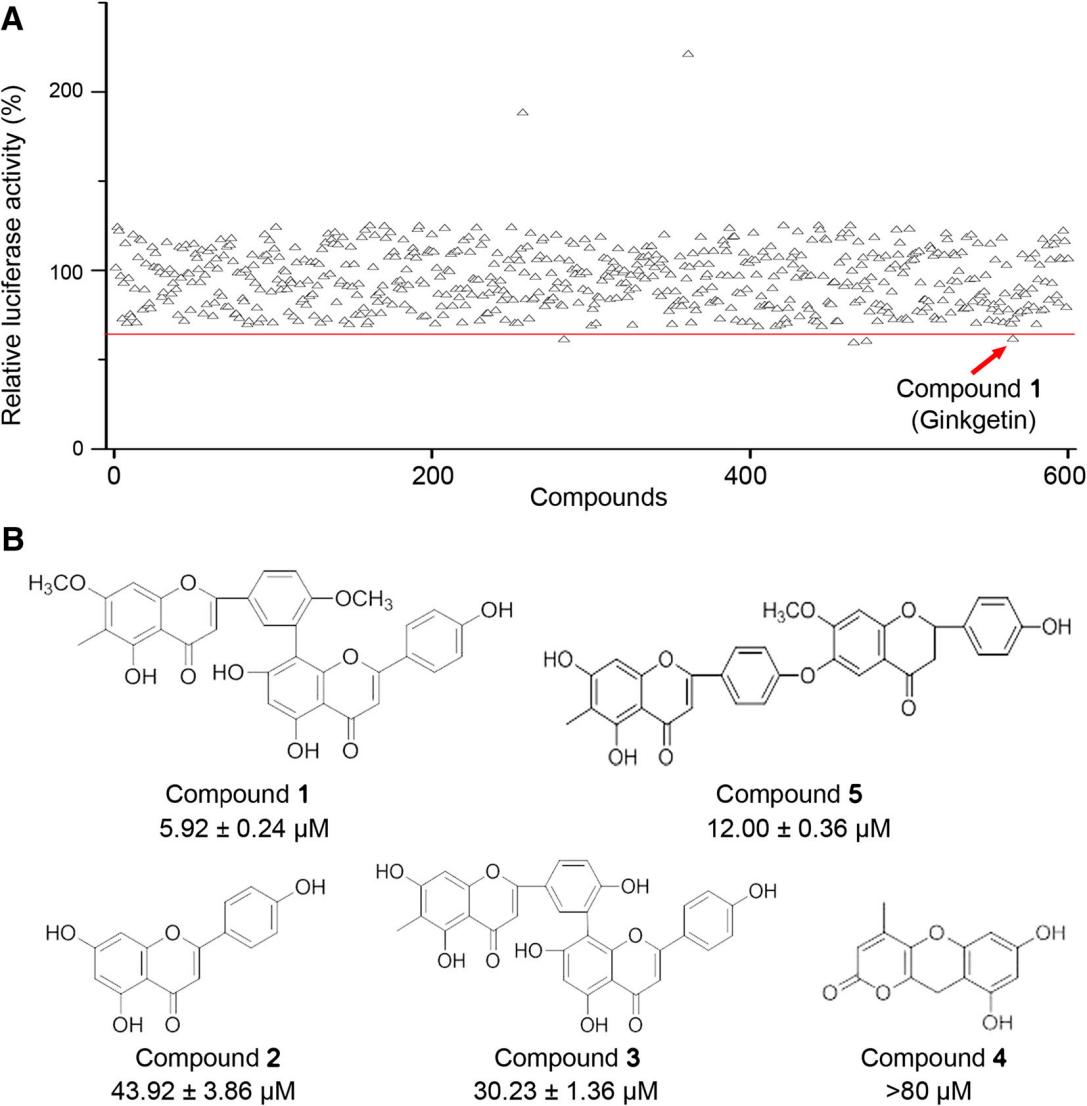
Screening for Wnt antagonists. **a** The HEK293W cells were treated with tested compounds for 24 h and relative luciferase activities (Topflash/Renilla) were analyzed. The testing concentration of the compounds was set based on the prior cytotoxicity of other cancer cell lines. The *red line* represents the 60 % relative Topflash/Renilla activity normalized to the control. **b** Structures of Ginkgetin and its derivatives and their IC_50_ values on Wnt pathway. (Color figure online)

To identify potent inhibitors of Wnt signaling, we conducted a primary screening in Wnt3a stably over-expressed HEK293W cells [24] with 600 compounds from library based on anti-tumor activity, stability and molecular mass. The initial screening concentration of each compound was set by referring to the cytotoxicity assessed primarily with other cancer cell lines. As shown in Fig. 1a, we identified four natural compounds that induced at least 40 % reduction of the relative luciferase activity without apparent cytotoxicity, giving a hit rate of 0.67 %. In addition, it was noteworthy that two Wnt signaling activators were identified in our screening, with relative luciferase activity 188 and 221 % compared to control, respectively.

In the following re-screening tests of the primary hits, the reporter cells were incubated with various concentrations of the compounds to estimate the potential inhibitory effects with IC_50_. Among the primary hits, a series of flavone derivatives exhibited inhibitory activity towards Wnt signaling pathway. In the secondary screening, compound **1** (Ginkgetin) exhibited the most potent inhibitory effect on Wnt signaling in a dose-dependent manner with a calculated IC_50_ of 5.92 ± 0.24 μΜ (Fig. 1b). The analogue compound **5** (Taiwanhomoflavone B) showed moderate inhibitory effect on Wnt signaling (12.00 ± 0.36 μΜ) (Fig. 1b), while the analogues, compounds **2**–**4**, namely Apigenin, Dedimethyene-taiwanhomoflavone A and 7,9-dihydroxy-4-methylpyrano[3,2-b]chromen-2(10*H*)-one, respectively, showed weak or even no effect on Wnt signaling (Fig. 1b). These compounds were isolated from *Cephalotaxus fortunei* var. *alpina*. The chemical structures of compounds **1**–**5** were shown in Fig. 1b and verified by the spectra data of MS and NMR [25–29].

According to the above data, the structure–activity relationship analysis within the five compounds suggested that the biflavone (compounds **1**, **3** and **5**) exhibited better inhibitory activity on the Wnt Signaling in comparison to flavone (compounds **2** and **4**). Moreover, the methoxy group substitutions of compound **1** (Ginkgetin) at position 7 and 4′ significantly improved the Wnt inhibitory potency compared to compound **3**, indicating that the skeleton of biflavone and the methoxy group substituted at position 7 and 4′ may be responsible for the inhibitory effect on Wnt signaling. To our knowledge, biflavones have not previously been identified as inhibitors of Wnt pathway, whereas the flavone has been reported as the antagonist [30] or activator [31] of Wnt pathway.

### Ginkgetin Inhibits the Cell Growth and Induces G_2_/M Cell Cycle Arrest in Medulloblastoma Cells

**Fig. 2:**
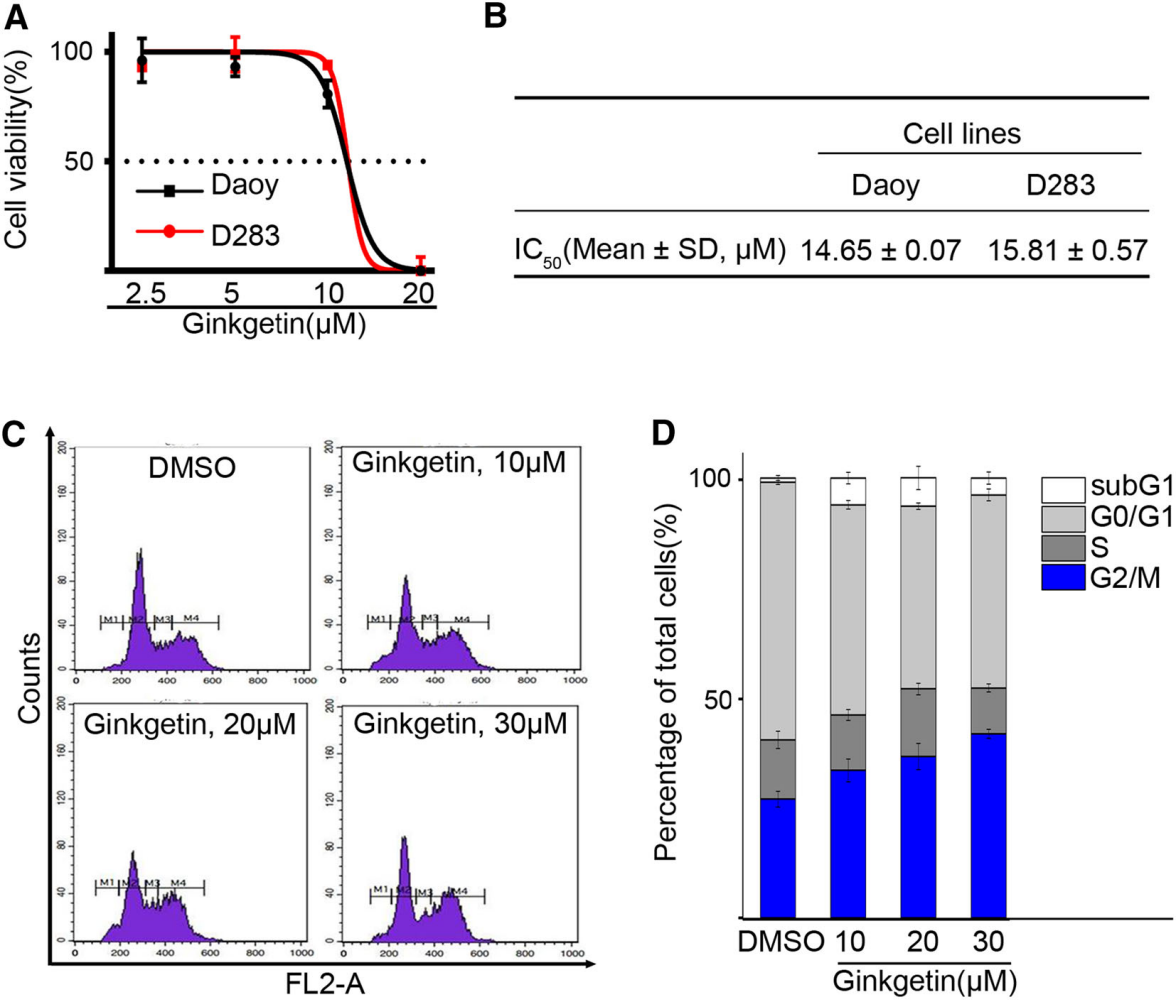
Ginkgetin inhibited the growth of Daoy and D283 cell lines, and induced G_2_/M cell cycle arrest in Daoy cells. **a** Effects of Ginkgetin on cell viability. Daoy and D283 cells were treated with Ginkgetin for 48 h. Cell viability was detected by MTS assay and represented with relative viability versus control. **b** Calculated IC_50_ values of the cytotoxicity of Ginkgetin (mean ± SD, n = 3). **c** Ginkgetin induced G_2_/M arrest in Daoy cells. Cells were incubated with Ginkgetin at indicated concentrations for 24 h. Then the cells were stained with PI (propidium iodide) and analyzed by flow cytometry. Counts of G_2_/M phase cells increased remarkably under Ginkgetin treatment in a dose-dependent manner. **d** Quantification of flow cytometry analysis of cell cycle (mean ± SD, n = 3)

As is known, the aberrant activation of Wnt pathway is correlated with sporadic MB [32], and literature data suggested *β*-catenin and other Wnt pathway components are over-activated in Daoy [33] and D283 [20, 34] cells. Thus the cytotoxicity of Ginkgetin was tested towards the two MB cell lines. Cells were exposed to the compound at concentrations up to 20 μM and the calculated IC_50_ values were 14.65 ± 0.07 and 15.81 ± 0.57 μM, towards Daoy and D283 cells respectively (Fig. 2a, b).

Wnt signaling regulates a succession of events involved in cell proliferation, differentiation and motility, especially cell cycle progress [35]. For example, the downstream target gene cyclinD1 is required for cell cycle progression in G_1_/S transition, and the pathway also participates in mitosis [36]. Therefore, we postulate that as a Wnt inhibitor, Ginkgetin suppressed cell proliferation possibly by regulating cell cycle. We then detected the effect of Ginkgetin on cell cycle in Daoy cells, as shown in Fig. 2c, d, the percentage of Ginkgetin treated cells at G_2_/M phase was increased, compared with that of control, indicating a G_2_/M cell phase arrest. In addition to being a target gene of the Wnt pathway, Axin2 is a scaffold protein involved in *β*-catenin inactivation, and locates at the mitotic spindle and the centrosomes to regulate the spindle checkpoint [37]. Giodini et al. [38]. demonstrated that survivin, another target gene of Wnt signaling, also functioned at cell division by controlling microtubule stability and assembly of mitotic spindle. Our results demonstrated that Ginkgetin, as a Wnt signaling antagonist, induced the G_2_/M arrest of Daoy MB cells, which might lead to the suppression of cell growth detected in the MTS assay.

### Ginkgetin Impairs Wnt Pathway in MB Cancer Cells Without Affecting the Expression of β-Catenin

**Fig. 3:**
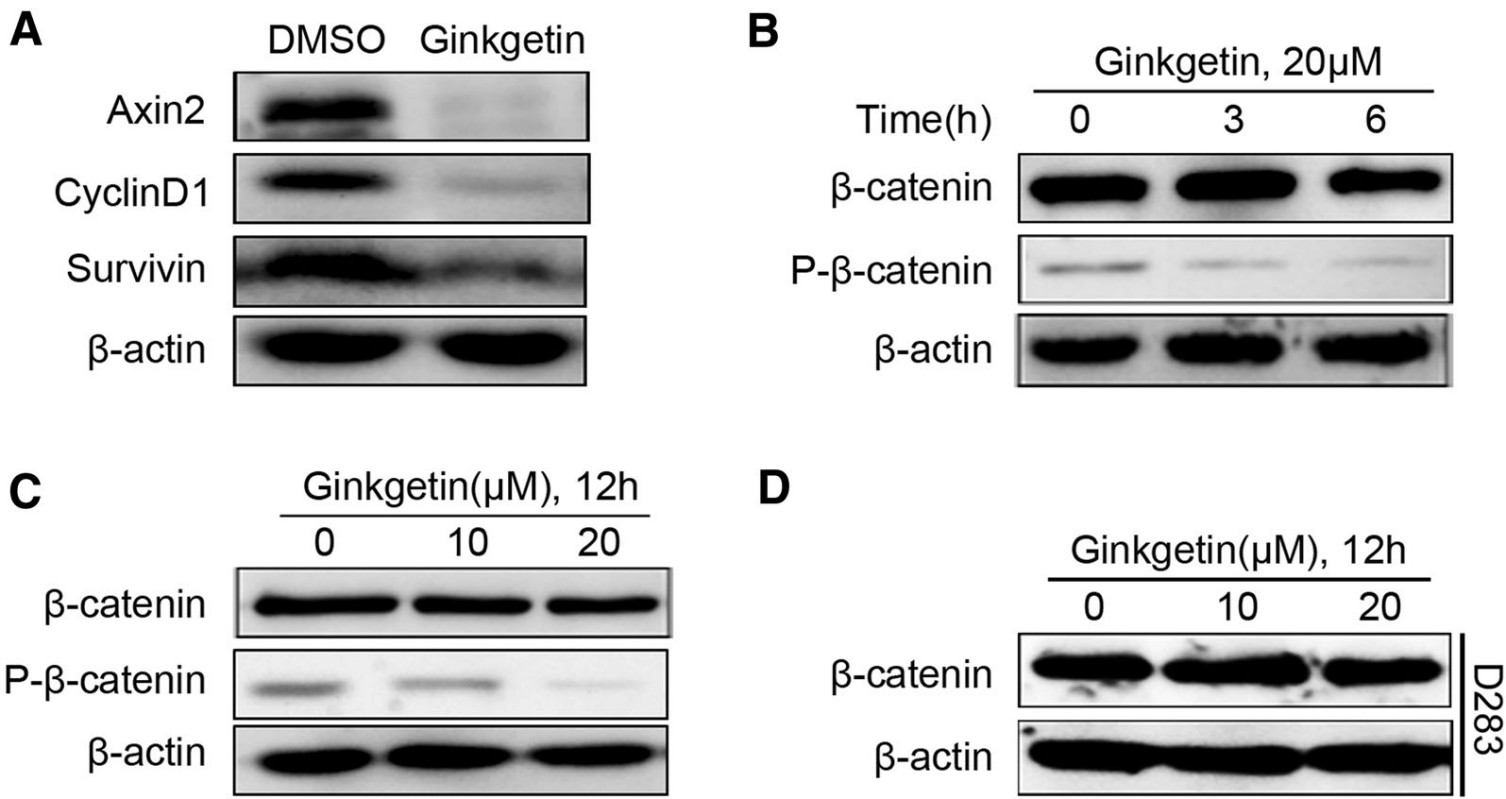
Ginkgetin down-regulated the expression of Wnt target genes without affecting the expression of *β*-catenin in MB cells. **a** The expression of Wnt target genes, Axin2, cyclin D1 and survivin, were suppressed by Ginkgetin at 20 μM for 24 h in Daoy cells. **b**, **c** The levels of total *β*-catenin remained unaffected by Ginkgetin in Daoy cells, with phosphorylated *β*-catenin (*p*-*β*-catenin) moderately diminished in a time- and concentration-dependent manner. **d** The total *β*-catenin level in D283 cells remained constant after Ginkgetin treatment. All the experiments were performed at least three times and the representative data were shown

Giving that Ginkgetin potently inhibited Wnt signaling, we next investigated whether it down-regulated Wnt signaling in MB cells, with the detection of the expression of the target genes, namely Axin2 [39], cyclinD1 [40] and survivin [41]. Western blotting analysis demonstrated that exposure of Daoy cells to 20 μM Ginkgetin for 24 h significantly attenuated the expression of Axin2, cyclinD1 and survivin (Fig. 3a). The expression suppression indicated that the anti-proliferation activity of Ginkgetin on MB attributed to the inhibition of Wnt signaling.

*β*-Catenin is the pivotal component in the Wnt pathway, and its accumulation in the cytoplasm will promote translocation into the nucleus, then binding to the TCF/LEF for transcriptional activation of downstream genes [42]. Since the *β*-catenin level is regulated by its phosphorylation and subsequent degradation, we next conducted western blot analysis to investigate whether Ginkgetin affected the *β*-catenin expression or its phosphorylation. Results showed that there was no obvious change of the level of total *β*-catenin in MB cells incubated with Ginkgetin (Fig. 3b–d), either in Daoy or in D283 cells, which indicated that Ginkgetin might regulate the Wnt signaling downstream of *β*-catenin. We observed a moderate reduction of phosphorylation at Ser 33/37/Thr 41 residues under Ginkgetin treatment in a time- and concentration-dependent manner (Fig. 3b, c), which was contrary to our expectation. These data indicated that Ginkgetin may inhibit Wnt signaling pathway downstream of *β*-catenin destruction. To provide an interpretation for this discrepancy, the distribution of *β*-catenin in the nuclear and cytoplasmic compartments, and the association of *β*-catenin with transcription factors TCF4/LEF1 need to be further investigated.

## Conclusion

We identified biflavone Ginkgetin as a small molecular inhibitor of Wnt pathway for the first time with a screening from 600 natural compounds. Structure–activity relationship was investigated to deduce the effective substituents. Ginkgetin exhibited potent cytotoxic activity and induced G_2_/M cell cycle arrest in MB cells. Inhibition of Ginkgetin on Wnt pathway and the preliminary mechanisms were investigated in Daoy and D283 cell lines. Further studies will provoke Ginkgetin as a promising candidate for anti-medulloblastoma drug development, thus providing novel therapeutic strategies for MB with minimum adverse effects.

## General Experimental Procedures

### Materials

Cell culture medium and fetal bovine serum (FBS) were provided by HyClone (Logan, Ut, USA). Daoy and D283 cells were obtained from Cell Research Center, IBMS, CAMS/PUMC. CellTiter 96^®^ AQueous One Solution Cell Proliferation Assay (MTS) and Dual-Luciferase^®^ Reporter (DLR™) Assay System were from Promega (Madison, WI, USA). Antibodies of β-actin, cyclinD1 and survivin were from Santa Cruz; anti-Axin2 and phospho-*β*-catenin (Ser33/37/Thr41) were from cell signaling technology; anti-*β*-catenin was from BD transduction laboratories. Rabbit anti-mouse IgG-Peroxidase and goat anti-rabbit IgG peroxidase were purchased from Sigma-Aldrich. Propidium iodide for cell cycle analysis was from Sigma-Aldrich. The library of natural compounds was supplied by the State Key laboratory of Phytochemistry and Plant resources in West China (Kunming Institute of Botany, Chinese Academy of Sciences, China).

### Cell Culture

Daoy cells were propagated in Dulbecco’s modified Eagle’s medium (DMEM), D283 cells were maintained in minimum essential medium (MEM), both supplemented with 10 % fetal bovine serum, 100 units/mL penicillin G sodium and 100 μg/mL streptomycin (HyClone). HEK293W cells were sustained in DMEM medium, supplemented with 10 % fetal bovine serum, 100 units/mL penicillin G sodium, 100 μg/mL streptomycin (HyClone), 100 μg/mL G418 and 100 μg/mL Hygromycin B. Cells were maintained with 5 % CO_2_ in a humidified incubator at 37 °C.

### Wnt Inhibitor Screening

The HEK293W cell line has been constructed in our lab as previously described [24, 43], and cells were seeded in 96 well plate and incubated overnight. The natural compounds from the State Key Laboratory of Phytochemistry and Plant Resources in West China with anti-tumour activity in DMSO stock solution were screened to identify novel small molecules inhibiting Wnt pathway. After 24 h treatment with compounds, the luciferase activities were measured using the Dual-Luciferase^®^ Reporter (DLR™) Assay System (Promega).

### Cell Proliferation Assay

Cells were seeded in 96-well plates (5 × 10^3^ cells/well) in triplicate and incubated for 12 h. Next, cells were exposed to the tested compounds with concentrations ranging from 2.5 to 20 μΜ for 48 h. Cell viability was measured by the MTS assay, according to the manufacturer’s protocol. The IC_50_ values (concentrations of 50 % inhibition of cell viability) were determined by the relative survival curve.

### Western Blot Analysis

Protein expression of *β*-catenin, phospho-*β*-catenin (Ser33/Ser37/Thr41), Axin2, cyclinD1 and survivin were detected by Western blotting. Cells were seeded into six-well plates and treated with Ginkgetin for 3 to 24 h. Cell extracts were separated by sodium dodecyl sulfate–polyacrylamide gel electrophoresis, transferred to polyvinylidene fluoride membranes (Millipore; Billerica, MA, USA) and then incubated with the following primary antibodies: rabbit anti-phospho-*β*-catenin(Ser33/Ser37/Thr41), rabbit anti-Axin2, mouse anti-*β*-catenin, mouse anti-cyclinD1, mouse anti-survivin, and mouse anti-*β*-actin as loading control. HRP conjugated anti-mouse IgG and anti-rabbit IgG were used as secondary antibodies. SuperSignal West Pico Chemiluminescent Substrate (Thermo scientific) was used to detect chemiluminescence, and blots were imaged using the Luminescent Image Analyzer LAS-4000mini System (GE, USA).

### Cell Cycle Analysis

Cell cycle was detected by flow cytometry with propidium iodide staining. Daoy cells were incubated with various concentrations of Ginkgetin, respectively, for 24 h. Cells were then collected and fixed with pre-cold 70 % ethanol overnight. Washed with PBS, fixed cells were digested with RNase (25 μg/mL) for 30 min at 37 °C, and then stained with PI (100 μg/mL). Fluorescence intensity was analyzed by FACSCalibur1 flow cytometer (BD Biosciences, San Jose, CA, USA). The percentages of the cells distributed in different phases of the cell cycle were determined using ModFIT LT 2.0.
